# Interleukin-33 Primes Mast Cells for Activation by IgG Immune Complexes

**DOI:** 10.1371/journal.pone.0047252

**Published:** 2012-10-11

**Authors:** Shinjiro Kaieda, Jun-Xia Wang, Ruslan Shnayder, Nadia Fishgal, Hillary Hei, Richard T. Lee, Richard L. Stevens, Peter A. Nigrovic

**Affiliations:** 1 Department of Medicine, Division of Rheumatology, Immunology, and Allergy, Brigham and Women’s Hospital, Harvard Medical School, Boston, Massachusetts, United States of America; 2 Department of Medicine, Division of Cardiovascular Medicine, Brigham and Women’s Hospital, Harvard Medical School, Boston, Massachusetts, United States of America; 3 Department of Medicine, Division of Immunology, Boston Children’s Hospital, Harvard Medical School, Boston, Massachusetts, United States of America; University of São Paulo, Brazil

## Abstract

Mast cells (MCs) are heterogeneous cells whose phenotype is modulated by signals received from the local microenvironment. Recent studies have identified the mesenchymal-derived cytokine IL-33 as a potent direct activator of MCs, as well as regulator of their effector phenotype, and have implicated this activity in the ability of mast cells to contribute to murine experimental arthritis. We explored the hypothesis that IL-33 enables participation of synovial MCs in murine K/BxN arthritis by promoting their activation by IgG immune complexes. Compared to wild-type (WT) control mice, transgenic animals lacking the IL-33 receptor ST2 exhibited impaired MC-dependent immune complex-induced vascular permeability (flare) and attenuated K/BxN arthritis. Whereas participation of MCs in this model is mediated by the activating IgG receptor FcγRIII, we pre-incubated bone marrow-derived MCs with IL-33 and found not only direct induction of cytokine release but also a marked increase in FcγRIII-driven production of critical arthritogenic mediators including IL-1β and CXCL2. This “priming” effect was associated with mRNA accumulation rather than altered expression of Fcγ receptors, could be mimicked by co-culture of WT but not ST2^−/−^ MCs with synovial fibroblasts, and was blocked by antibodies against IL-33. In turn, WT but not ST2^−/−^ MCs augmented fibroblast expression of IL-33, forming a positive feedback circuit. Together, these findings confirm a novel role for IL-33 as an amplifier of IgG immune complex-mediated inflammation and identify a potential MC-fibroblast amplification loop dependent on IL-33 and ST2.

## Introduction

Mast cells (MCs) are hematopoietic cells that develop from circulating progenitors and differentiate into fully granulated effector cells within the local tissue milieu. The survival, development, phenotype, and function of these immune cells are modulated by contact-dependent and -independent signals from the microenvironment [1], [2], [3], [4]. In many locations, MCs reside in close proximity to fibroblasts, and prior work has demonstrated the existence of intricate physical contacts between the two cell types [5], [6], [7]. This interaction is of critical importance to MCs, since expression of membrane-bound Kit ligand (KitL) by fibroblasts enables the survival of MCs in tissues [8], [9]. Fibroblasts also modulate the effector phenotype of MCs, including their expression of eicosanoids and granule proteases [5], [6]. In turn, MCs influence the growth and activation of fibroblasts [10], [11].

Among the multiple factors known to influence MC phenotype and behavior, recent interest has focused on IL-33, a pro-inflammatory member of the IL-1 cytokine family [12], [13]. IL-33 is produced primarily by fibroblasts, smooth muscle cells, keratinocytes, and endothelial cells; MCs themselves have also been identified as a potential source [14], [15]. Acting via its receptor ST2, IL-33 triggers MCs to release numerous cytokines and chemokines [16], [17], [18], [19], [20], [21], [22], an activity implicated in the pathogenesis of anaphylaxis and in the role of mast cells as sensors of tissue injury [22], [23]. Moreover, exposure of MCs to IL-33 augments expression of cytokines in MCs activated concomitantly via the high-affinity IgE receptor FcεRI [24], [25]. IL-33 is also the first factor shown to promote the accumulation in granules of mouse MC protease 6 [26], an ortholog of human tryptase β that plays a role in innate immunity and inflammatory arthritis [27], [28], [29]. Thus, IL-33 also is a granule maturation factor for MCs.

Recent studies have implicated IL-33 in the activation of synovial MCs in murine arthritis [21], [30], [31]. Transgenic mice lacking ST2 exhibit impaired degranulation of synovial MCs, while MCs cultured overnight in the presence of arthritogenic K/BxN mouse serum have been reported to become susceptible to IL-33-induced degranulation [31]. However, MC-dependent vasogenic edema begins within minutes of the administration of K/BxN mouse serum, a time course that may be too rapid for *de novo* release of IL-33 [14], [32], [33], [34]. Further, genetic studies have demonstrated that FcγRIII is an obligate pathway for the activation of synovial MCs in K/BxN arthritis [33], [35]. It therefore remains unclear, in the context of arthritis, whether the major role of IL-33 is to activate MCs directly or rather to potentiate their activation via other pathways, such as Fc receptors. To address this question, we tested the role of IL-33 in the response of MCs to FcγRIII ligation, and explored the role of this cytokine in the interactions between MCs and synovial fibroblasts, a potential source of IL-33 within the joint.

## Materials and Methods

### Reagents

The Mouse Cytokine Array Panel A, recombinant mouse IL-33, and specific ELISAs for CXCL2, tumor necrosis factor-α (TNF-α), IL-1β, IL-6, IL-33 and anti-IL-33 Ab (MAB3626) were obtained from R&D Systems (Minneapolis, MN). Mouse IgG1 isotype antibody was from Biolegend (San Diego, CA). Anti-IL-33 (Nessy-1) was from Enzo Life Sciences (Farmingdale, NY). Recombinant mouse IL-3 and KitL were obtained from Peprotech (Rocky Hill, NJ), and endotoxin-free rat anti-mouse FcγRII/III antibody (Ab) (clone 2.4G2) was obtained from BioXCell (West Lebanon, NH). Additional reagents included 2.4G2-PE Ab (BD Biosciences, San Diego, CA) and rat anti-FcγRIII Abs (clone 275003, unconjugated and carboxyfluorescein conjugated, R&D Systems).

### Mice

Wild-type (WT) C57BL/6J (B6) and B6;129S4-Fcγr2b^tm1Ttk^/J (FcγRII^−/−^) mice were from the Jackson Labs (Bar Harbor, ME). ST2^−/−^ B6 mice were obtained from Dr. Andrew McKenzie (MRC Laboratory of Molecular Biology) [36]. Animal experiments were approved by the Institutional Animal Care and Use Committee of the Dana Farber Cancer Institute (Animal Welfare Assurance Number: A3023-01). All efforts were made to minimize the suffering of animals used in this research.

### Experimental Arthritis

Pro-arthritic serum was isolated from K/BxN mice as previously described [37]. K/BxN arthritis was induced by the intraperitoneal injection of diluted K/BxN serum (75 µl serum with 75 µl endotoxin-free PBS) on days 0 and 2 of each experiment. Arthritis was graded using a 0–12 clinical scale (0–3 per paw) as well as by caliper measurement of ankle thickness, as described [37]. “Flare” (acute paw swelling) was measured by caliper in all 4 paws 30 minutes after the first serum administration [33]. Histological assessment was performed on paraffin-embedded 4-µm sections stained with hematoxylin and eosin, and synovial inflammation, cartilage injury, and bone erosion were graded in blinded fashion on a 0–5 scale using an established system [35].

### Cell Culture and Mast Cell Activation

Mouse bone marrow derived MCs (mBMMCs) were developed by culturing bone marrow cells for at least 4 weeks in 10% FBS DMEM media supplemented with IL-3 (10 ng/ml) and KitL (25 ng/ml), as previously described [37]. Fibroblast-like synoviocytes (FLS) were cultured from collagenase-digested mouse ankles in 10% FBS DMEM media [38]. For co-culture experiments where FLS are a potential source of KitL, mBMMCs were derived as above but using only IL-3 (10 ng/ml) [39]. Co-culture of IL-3-developed mBMMCs and FLS was performed in the presence of 10 ng/ml recombinant IL-3, with the cell types separated by a membrane containing 0.2 µm pores [26]. Activation of IL-3/KitL-developed mBMMCs via FcγRIII was achieved by low-speed centrifugation against plate-bound 2.4G2 Ab as described [35]. For these experiments, cells were maintained in their pre-incubation medium (i.e., containing IL-33 if added, as well as any mediators released through the action of IL-33 prior to crosslinking of FcγRIII by plate-bound 2.4G2). Quantification of cytokine array spot density for the Mouse Cytokine Array Panel A was performed using NIH ImageJ and normalized to positive controls in each membrane as per manufacturer instructions.

### Real-time Quantitative PCR (qPCR)

Total RNA was isolated from mBMMCs, FLS, and ankle joints using the RNeasy minikit (Qiagen, Valencia, CA), followed by qPCR employing primers specific for IL-6, IL-1β, TNF-α, IL-33, CXCL2, β-Actin and glyceraldehyde 3-phosphate dehydrogenase (GAPDH) [26], [33], [40].

### Statistical Analysis

*P* values were calculated by Student’s t-test, where normality was confirmed using the Kolmogorov-Smirnov test, by Mann-Whitney for non-parametric data, and by ANOVA for curves (GraphPad Prism, version 4.0). Synergistic interaction between stimuli was evaluated using linear regression (SAS, version 9.2). *P* values smaller than 0.05 were considered significant.

## Results

### ST2 Promotes Disease Severity and Mast Cell Activation in K/BxN Arthritis

**Figure 1:**
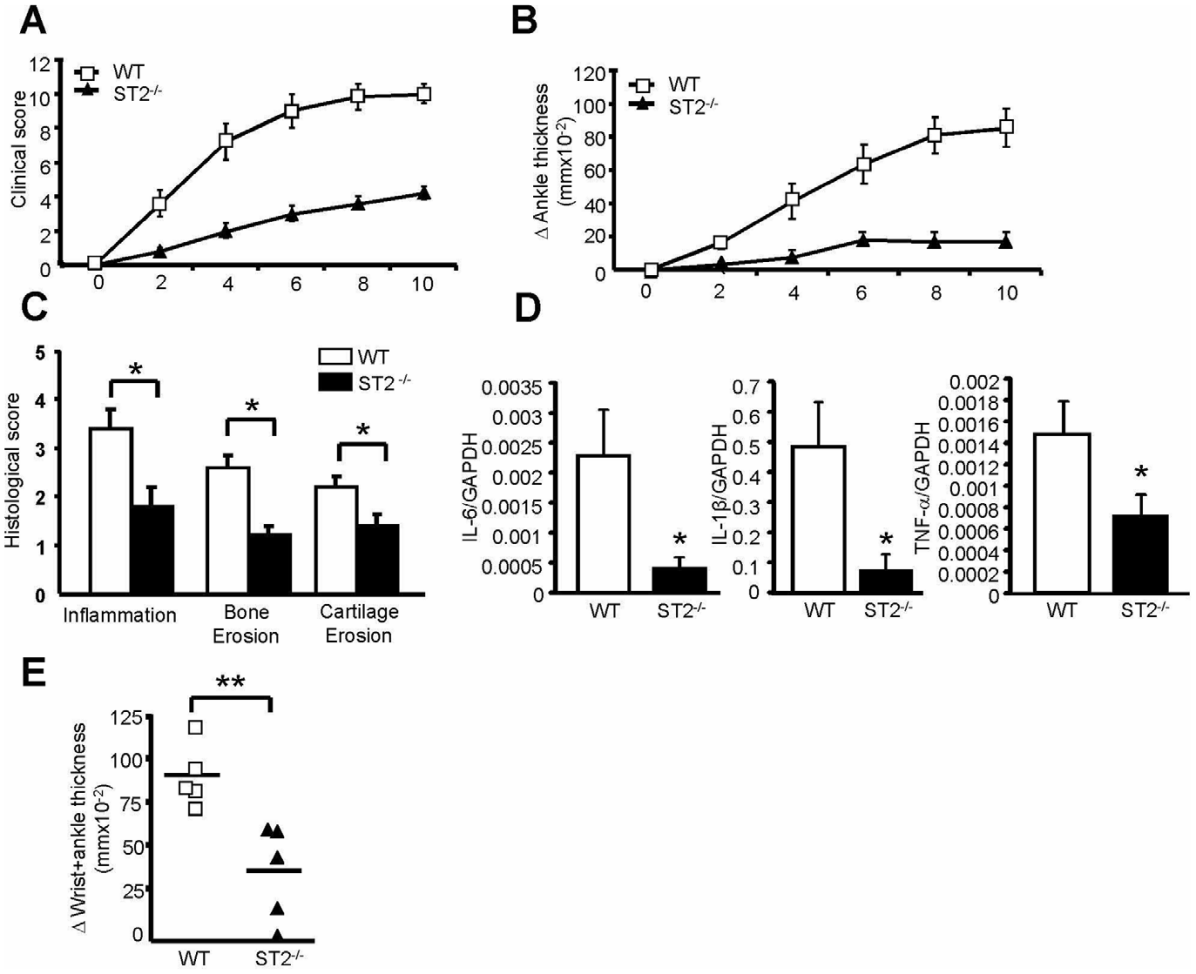
ST2 deficiency attenuates K/BxN arthritis. Arthritis was initiated in ST2^−/−^ mice and their WT littermates via intraperitoneal administration of K/BxN mouse serum on days 0 and 2 (n = 5/group). (A) Clinical score on a 0–12 scale, *P*<0.0001, WT versus ST2^−/−^. (B) Change in ankle thickness, *P*<0.0001, WT versus ST2^−/−^. (C) Histomorphometric quantification of arthritic tissue (5 ankles/group). (D) Cytokine mRNA in ankle lysates (10 ankles/group from two separate experiments) at day 8 or 10 arthritis. (E) Acute change in wrist and ankle thickness (“flare”) measured 30 minutes after initial serum administration (n = 5/group). Results shown are the mean ± SEM. Panels A–C&E reflect 1 of 2 experiments with similar results. *P<0.05, **P<0.01, WT versus ST2^−/−^.

To explore the role of IL-33 in K/BxN arthritis and to evaluate early immune complex-mediated activation of MCs, we induced arthritis in mice lacking ST2 as well as in WT control mice. In agreement with published data [31], we found that ST2^−/−^ animals exhibited less severe arthritis (Figures 1A and 1B). Correspondingly, histological measures of arthritis were reduced, as was tissue expression of IL-6, IL-1β and TNF-α (Figures 1C and 1D). Congruent with the prior demonstration in arthritic day 4 tissues that MC degranulation is impaired by ST2 deficiency [31], we found that acute MC-dependent vascular edema (“flare”) [33] was reduced in the transgenic mice 30 minutes after serum administration (Figure 1E). These results confirm the importance of ST2 in this model, including in initial MC activation.

### IL-33 Amplifies FcγRIII-mediated Mast Cell Mediator Production

**Figure 2:**
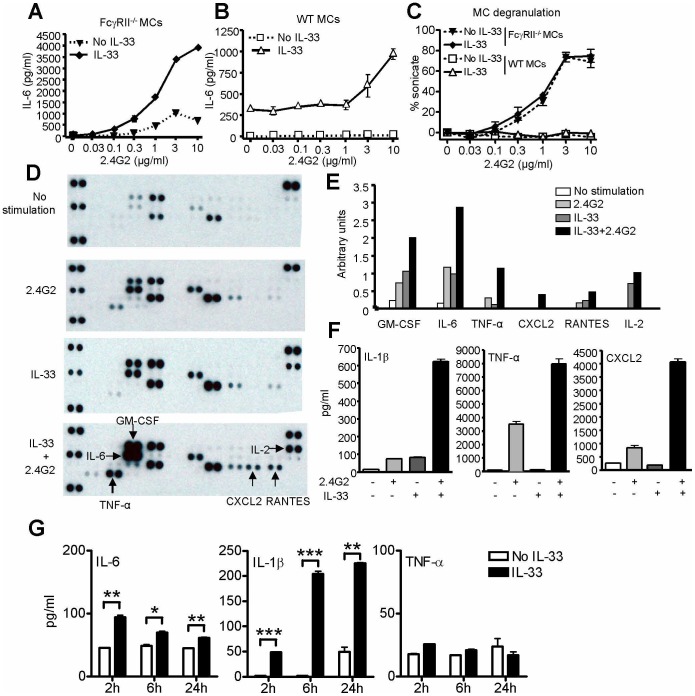
IL-33 enhances FcγRIII-mediated cytokine production by mast cells. (A–C) FcγRII^−/−^ or WT B6 mBMMCs were pre-incubated with or without IL-33 (10 ng/ml) for 4 hours, and then cells in pre-incubation media were spun onto plates pre-coated with anti-FcγRII/III Ab (2.4G2). Supernatants were harvested at 16 hours and assayed for IL-6 (A&B) and the granule mediator β-hexosaminidase (C). Differences in baseline IL-6 production were reproducibly observed between FcγRII^−/−^ and B6 mBMMCs, and may reflect divergent genetic backgrounds or other factors. (D) FcγRII^−/−^ mBMMCs incubated with or without IL-33 for 4 hours were activated by plate-bound 2.4G2 Ab (10 µg/ml) for 16 hours and assayed by multiplex cytokine array. (E) Quantitation of optical density of selected mediators from D (mean of 2 dots). (F) Assay of mediators identified in D-E via specific ELISA in separate experiments employing an identical experimental design (IL-1β performed on lysates). (G) To determine whether IL-33 induced intracellular accumulation of cytokine, B6 mBMMCs were stimulated with IL-33 (10 ng/ml) for the intervals indicated, washed×2 in ice-cold PBS, and lysed in the presence of protease inhibitors. All results representative of at least 2 independent experiments. *P<0.05, **P<0.01, ***P<0.001.

FcγRIII and FcγRII are stimulatory and inhibitory IgG receptors, respectively, that mediate opposing effects on immune complex-induced MC activation. In K/BxN arthritis, engraftment experiments have shown that effective engagement of synovial MCs requires the activating IgG receptor FcγRIII [35]. Further, synovial MCs lacking FcγRIII fail to degranulate upon serum administration [33]. Expression of the C5a complement receptor CD88 by MCs is also required, though at a step downstream of degranulation [33]. To assess whether IL-33 enhances MC activation via FcγRIII, we employed an established *in vitro* system [35]. WT mBMMCs and transgenic mBMMCs lacking FcγRII were cultured for 4 hours in the presence or absence of IL-33. The resulting cells were then activated via plate-bound anti-FcγRII/III Ab (clone 2.4G2). IL-33 markedly amplified IL-6 production by FcγRII^−/−^ cells (Figure 2A). Interestingly, whereas activation of WT mBMMCs by 2.4G2 is typically blocked due to engagement of the inhibitory receptor FcγRII, IL-33 enabled MC to partially bypass this blockade and respond productively to this immune complex mimic (Figure 2B). Of note, we failed to observe any effect of IL-33 on exocytosis of the granule constituent β-hexosaminidase (Figure 2C), a defect that could not be reversed using published methods [23], [31], including overnight pre-incubation with IgE (anti-trinitrophenyl, 0.3 µg/ml) or K/BxN mouse serum (1∶100-1∶1) (data not shown). We also could not identify a synergistic effect of exogenous IL-33 and C5a in our mBMMC, with or without concomitant FcγRII/III ligation (data not shown).

To understand more fully the effect of cooperative IL-33- and FcγRIII-mediated activation of MCs, we employed a multiplex array to evaluate cytokines and chemokines elaborated by 2.4G2-stimulated FcγRII^−/−^ mBMMCs, pre-incubated with IL-33 or vehicle for 4 hours. The results confirmed our IL-6 data, and showed that amplification extended to other mediators as well, most prominently TNF-α and CXCL2 (Figure 2D, quantitated in Figure 2E). The latter finding is particularly interesting, since arthritis is neutrophil dependent and the receptor CXCR2 has been implicated in IL-33-mediated neutrophil recruitment [41], [42], [43]. Selected results were further confirmed by specific ELISAs (Figure 2F). Whereas IL-1β has been shown to be a key MC-derived arthritogenic cytokine [35], we assayed for IL-1β in lysates of FcγRIII-stimulated FcγRII^−/−^ mBMMCs and found a marked increase after IL-33 pre-incubation (Figure 2F). Linear regression demonstrated that the effect of sequential stimulation by IL-33 followed by FcγRIII ligation far exceeded an additive effect, confirming synergy (TNF-α *P* = 0.0005, IL-1β and CXCL2 *P*<0.0001). Interestingly, in mBMMCs stimulated with IL-33 and subsequently washed to remove released cytokine, accumulation of intracellular cytokine was observed for IL-6 and IL-1β, but not TNF-α (Figure 2G).

### Mast Cell Priming by IL-33 Reflects mRNA Accumulation Rather than FcγR Expression

**Figure 3:**
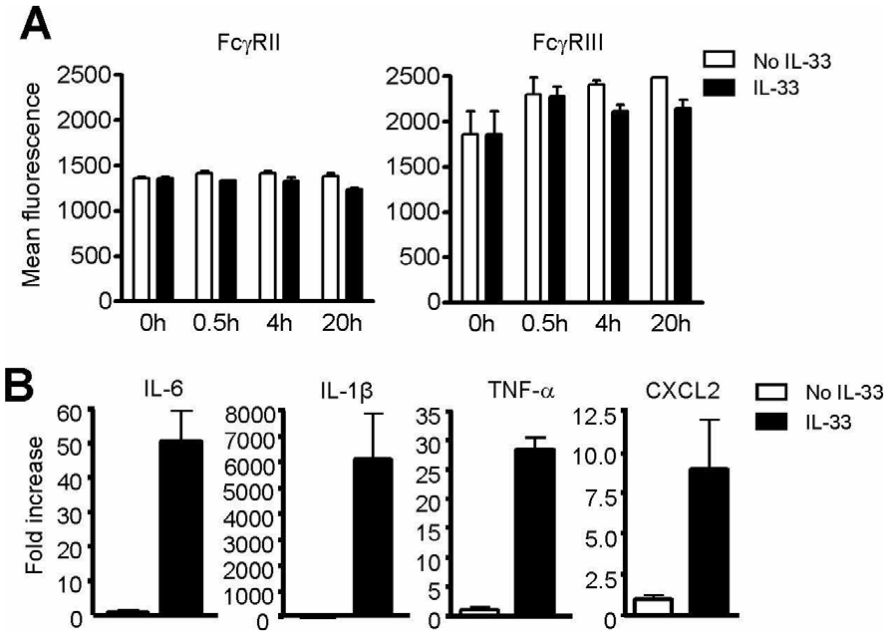
IL-33 priming of mast cells reflects accumulation of pro-inflammatory mRNA. (A) B6 mBMMCs were incubated with IL-33 (10 ng/ml) and analyzed for surface expression of inhibitory (FcγRII) and stimulatory (FcγRIII) IgG receptors by flow cytometry at the indicated time points. (B) Cytokine mRNA expression in B6 mBMMCs exposed to IL-33 (10 ng/ml) for 4 hours. Results reflect at least 2 independent experiments.

Exploring the mechanism of the observed synergy, we examined whether IL-33 pre-incubation altered FcγR expression and/or downstream processes involved in the expression, biosynthesis, and release of mediators. Reciprocal modulation of FcγRII and FcγRIII expression is a well-recognized pathway for enhancing the responsiveness of cells to immune complexes [44], although we have been unable to confirm that this mechanism is active in either mouse or human MCs [33]. Exposure of mBMMCs to IL-33 failed to alter surface expression of FcγRII or FcγRIII (Figure 3A), consistent with the lack of an effect of IL-33 on FcR-mediated MC activation threshold (Figure 2C). Rather, pre-incubation of mBMMCs with IL-33 induced a marked accumulation of transcripts that encode numerous pro-inflammatory factors. Most remarkably, the level of IL-1β transcript increased several hundred-fold, an effect that could be observed as low as 3–10 pg/ml of IL-33 (Figure 3B and data not shown). We interpret these results to indicate “priming” of MCs by IL-33, whereby exposure of MC to IL-33 alters the state of the cells to enable markedly enhanced production of pro-inflammatory mediators upon subsequent stimulation via FcγRIII.

### IL-33 and ST2 Mediate Mast Cell Priming by Fibroblasts

**Figure 4:**
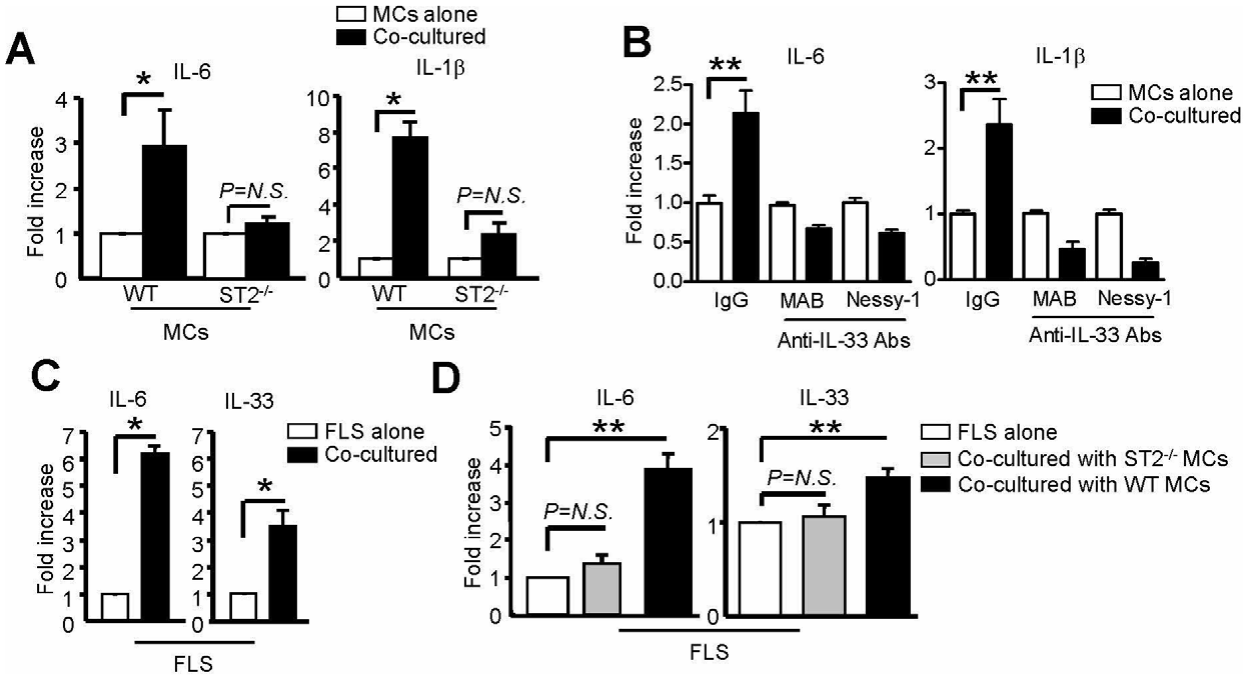
IL-33 mediates a MC-FLS amplification loop. MCs were cultured with or without FLS for 1–2 weeks in the upper and lower chambers, respectively, of a transwell apparatus. (A) Cytokine mRNA expression in WT and ST2^−/−^ MCs after co-culture with FLS. (B) Cytokine mRNA levels in MCs with or without anti-IL-33 Ab treatment (10 µg/ml q4d). Data represent 2 independent experiments with similar results. (C–D) Cytokine mRNA levels in FLS. n = 2 wells per condition, reflective of 2–5 pooled experiments. *P<0.05, **P<0.01, N.S., not significant.

Whereas IL-33 may be elaborated by synovial fibroblasts [21], [30], we explored the possibility that this cytokine could be pivotal for MC-fibroblast interactions. For these experiments, we co-cultured mBMMCs and FLS in an established transwell system that prohibited direct cell-cell contact and permitted separate analysis of each cell type [26]. We found that FLS induced mBMMCs to increase their levels of transcripts for IL-6 and IL-1β. This effect was completely abrogated in ST2-deficient MCs (Figure 4A). Whereas IL-33 is potent at low concentrations and unstable to manipulation, we were unable to measure this cytokine consistently in our extended co-culture supernatants (detection limit 10–15 pg/ml). Therefore, we repeated our studies in the presence of blocking antibodies against IL-33 [45]. Consistent with our ST2^−/−^ findings, specific IL-33 antagonism abrogated mRNA accumulation in WT MCs (Figure 4B). These results implicate IL-33, acting via ST2, as the key soluble mediator driving accumulation of pro-inflammatory cytokine mRNA in MC in our co-culture system.

Interestingly, mBMMCs induced co-cultured FLS to increase their expression of IL-33 and IL-6 (Figure 4C). This reciprocal effect on FLS also required MCs to express ST2 (Figure 4D), indicating an ST2-dependent MC-FLS pro-inflammatory loop. Whereas MCs have recently been identified as a potential source of IL-33 [15], we assessed IL-33 mRNA from co-cultured mBMMCs in two experiments and found it to be either low (<0.03 vs. GAPDH) or absent (<0.0002 vs. GAPDH), indicating that FLS are the most likely source of IL-33 in our system. Of note, neutralizing antibodies against IL-6 and IL-1β failed to abrogate the loop (data not shown). Therefore, the identity of the MC-derived soluble factor(s) mediating IL-33 mRNA up-regulation in FLS remains to be determined.

## Discussion

Among their many functions, MCs are immune sentinels, residing near epithelial surfaces, blood vessels, and near vulnerable body cavities where they serve to provide surveillance against pathogen invasion, tissue injury, and other insults [13], [46]. Upon activation, MCs can elaborate a range of responses depending not only upon the stimulus but also upon their particular phenotype [4]. MCs from different tissue sites express distinct surface receptors, intracellular proteases, and other effector molecules. These phenotypic changes are mediated by the local environment, though the detailed pathways involved are incompletely defined.

**Figure 5:**
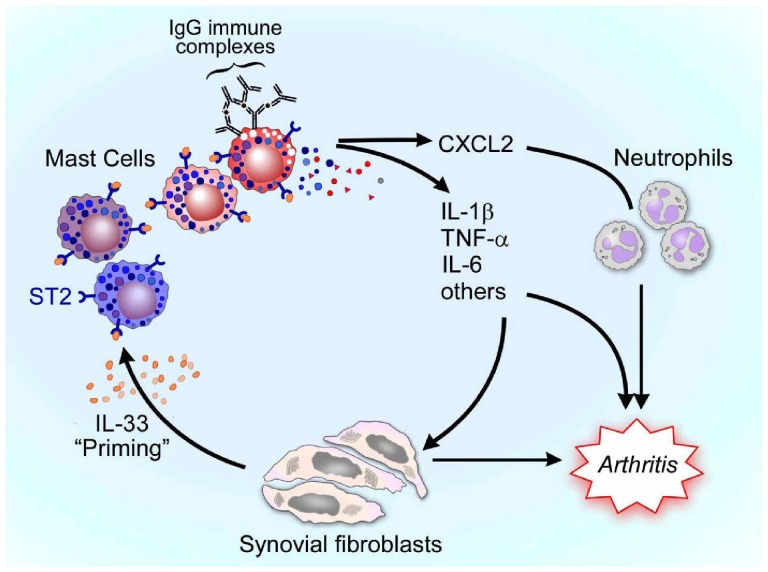
IL-33-mediated priming of MCs for immune complex-dependent arthritis. In the model proposed, synovial fibroblasts release IL-33 in a constitutive or induced manner. IL-33 causes phenotypic changes in neighboring MCs, including accumulation of cytokine mRNA and alteration in granule content, depicted as color change in “primed” MC. Upon exposure to immune complexes, primed MCs exhibit release pro-inflammatory mediators that further activate fibroblasts, promote neutrophil recruitment, and contribute to arthritis severity. Reciprocal signals from MCs stimulated via ST2 enhance IL-33 production by fibroblasts, constituting a MC-fibroblast amplification loop.

Here, we identify a new role for IL-33 and its receptor ST2 in IgG-mediated MC activation. We previously showed that MCs activated via FcγRIII elaborate IL-1β, and that this pathway is required for MCs to “jump start” IgG-mediated K/BxN murine arthritis [35]. However, the quantity of IL-1β found to be elaborated by cultured MCs stimulated *in vitro* via FcγRIII was smaller than might have been expected given the prominent *in vivo* role of this cytokine. The present work helps to bridge this gap. We now show that exposure of MCs to IL-33 dramatically increased their production of IL-1β upon FcγRIII ligation, and that this effect could be mimicked by co-culture with primary fibroblasts derived from mouse synovium. Further, we found that this relationship was reciprocal, in that MCs activated via ST2 augmented IL-33 expression in co-cultured FLS, representing a potential amplification loop (Figure 5). The *in vivo* importance of these *in vitro* observations was suggested by the reduced intensity of K/BxN arthritis in ST2^−/−^ mice, a phenotype associated by others with altered MC activation [31].

Beyond IL-1β, we found that IL-33 priming of MCs enhanced FcγRIII-mediated release of multiple other mediators, including CXCL2. The CXCL2 receptor CXCR2 is required for the development of full pathology in K/BxN arthritis, and in particular for neutrophil recruitment [29], [41], [43], [47]. Our findings therefore develop further our understanding of mechanisms by which MCs help recruit neutrophils to the inflamed joint [33].

These results are consistent with published data showing that IL-33 amplifies mediator production resulting from stimulation of MCs via IgE, since both FcγRIII and FcεRI signal via a common Fc receptor γ chain [24], [25]. Further, our results confirm that IL-33 can itself drive MC production of a range of mediators, including the unusual combination of IL-6 and IL-2 that could potentially contribute to the subversion of regulatory T cell function (Figure 2D) [48]. Such direct stimulation of MCs may contribute to inflammatory arthritis, particularly in chronic synovitis, when the population of synovial MCs is often markedly expanded [11].

However, our results suggest an alternate mechanism by which IL-33 contributes to acute MC activation in IgG-mediated arthritis. In K/BxN arthritis, the MC-dependent “flare” begins within minutes of serum administration, a timeframe probably too short for *de novo* IL-33 synthesis. Rather, consistent with published results demonstrating the key role of FcγRIII in synovial MC activation [33], [35], our data suggest that constitutive signals mediated via IL-33 promote immune complex responsiveness of synovial MCs, defining therefore a new model for a permissive role of IL-33 in MC-dependent immune complex disease (Figure 5). Whereas IL-33 pre-incubation induces accumulation of mRNA (and to a lesser extent intracellular protein) for key pro-inflammatory cytokines whose production by subsequent FcγRIII ligation is markedly enhanced, we hypothesize that such “pre-loading” of MC by IL-33 represents an important component of the priming mechanism, though other factors may also be involved.

Our results also expand appreciation of the integral relationship between MCs and fibroblasts. We previously demonstrated a profound effect of fibroblasts on the development of MCs [5], [6], [26]. The current work builds upon these studies, showing that IL-33 is a key mediator by which fibroblasts prime MCs for activation by IgG immune complexes. Given the known anatomic and functional associations of synovial MC with fibroblasts, these cells represent the most likely source of IL-33 in the joint, a possibility modeled by our *in vitro* co-culture system. However, endothelial cells or other IL-33-producing lineages, including MCs themselves, could potentially fulfill the same role.

While our *in vitro* findings correspond well to the expected activity of MCs in arthritis, it is possible that our system fails to model all aspects of the *in vivo* biology. In particular, we observed evidence for reduced MC activation in ST2^−/−^ animals exposed to K/BxN IgG, manifested as reduced flare magnitude. This result supports the observation that MC degranulation (observed at day 4 tissue harvest) is impaired in ST2^−/−^ mice administered K/BxN serum [31]. However, consistent with most published reports, we found no *in vitro* effect of IL-33 on degranulation of cultured MCs, either alone or together with FcγRIII ligation [13], [25]. Further, whereas exposure of WT MCs to IL-33 enabled these cells to bypass inhibition by FcγRII with respect to production of IL-6, we could not induce FcγRIII-mediated degranulation or IL-1β production (data not shown). These observations may reflect phenotypic variance between cultured MCs and those that have matured within synovial tissues, or potentially the absence of a required cofactor, given the recent finding that animals deficient in the receptor for IL-4 fail to demonstrate tissue MC degranulation induced by repeated injections of recombinant IL-33 [49]. Alternately, it may be that the initial MC activation step that provides the “jump start” to arthritis does not obligatorily involve degranulation. Indeed, the precise mechanisms mediating the flare remain to be defined, and are known to involve neutrophils as well as MCs, such that the flare is unlikely to simply represent local anaphylaxis-like release of MC granule contents [32], [50].

Our results do not define the pathways by which fibroblasts produce and release IL-33. Indeed, this remains an area of substantial uncertainty within IL-33 biology [34]. Like most other members of the IL-1 family, IL-33 does not possess a signal peptide permitting conventional secretion. Since IL-33 is inactivated by caspases, it has been suggested that it may represent a “alarmin,” liberated during necrosis but not apoptosis [51]. Indeed, some degree of necrosis was detectable in our cultures by lactate dehydrogenase release (data not shown), though whether such necrosis is relevant to our *in vitro* observation, or indeed to the *in vivo* arthritis phenotype, is unknown. Interestingly, we recently showed that cardiac fibroblasts can release IL-33 upon mechanical stretch, providing one potential mechanism by which fibroblasts within a moving joint might release IL-33, thereby priming MCs [52]. However, this mechanism would not have been expected to be operative in our static culture system.

In summary, our results show that IL-33 has the previously unrecognized potential to enhance MC responses to FcγRIII ligation. Our previous studies have demonstrated that MCs activated via FcγRIII can “jump start” synovial inflammation, at least in part via the pro-inflammatory cytokine IL-1β [35]. Recent *in vivo* studies, confirmed here, have implicated MC expression of ST2 in arthritis [21], [31]. Our current results link these observations together, showing that priming of MCs via IL-33 potentiates their activation via FcγRIII, resulting in markedly enhanced production of IL-1β, IL-6, and other mediators (Figure 5). Since immune complexes deposited within synovial tissue are a hallmark of rheumatoid arthritis [53], our results suggest that blockade of the IL-33/ST2 axis could benefit from a multiplier effect, dampening cell activation resulting not only from IL-33 itself but also from mechanisms amplified by this cytokine, including Fc receptors ligation in MCs. These results therefore further support IL-33 as a potential candidate for therapeutic inhibition in arthritis.

## References

[1] Nakano T, Sonoda T, Hayashi C, Yamatodani A, Kanayama Y, et al (1985) Fate of bone marrow-derived cultured mast cells after intracutaneous, intraperitoneal, and intravenous transfer into genetically mast cell-deficient W/Wv mice. Evidence that cultured mast cells can give rise to both connective tissue type and mucosal mast cells. J Exp Med 162: 1025–1043. 3897446 10.1084/jem.162.3.1025 PMC2187813

[2] Sonoda S, Sonoda T, Nakano T, Kanayama Y, Kanakura Y, et al (1986) Development of mucosal mast cells after injection of a single connective tissue-type mast cell in the stomach mucosa of genetically mast cell-deficient W/Wv mice. J Immunol 137: 1319–1322. 3734457

[3] Gurish MF, Pear WS, Stevens RL, Scott ML, Sokol K, et al (1995) Tissue-regulated differentiation and maturation of a v-abl-immortalized mast cell-committed progenitor. Immunity 3: 175–186. 7648391 10.1016/1074-7613(95)90087-x

[4] Galli SJ, Kalesnikoff J, Grimbaldeston MA, Piliponsky AM, Williams CM, et al (2005) Mast cells as “tunable” effector and immunoregulatory cells: recent advances. Annu Rev Immunol 23: 749–786. 15771585 10.1146/annurev.immunol.21.120601.141025

[5] Levi-Schaffer F, Austen KF, Gravallese PM, Stevens RL (1986) Coculture of interleukin 3-dependent mouse mast cells with fibroblasts results in a phenotypic change of the mast cells. Proc Natl Acad Sci U S A 83: 6485–6488. 3462707 10.1073/pnas.83.17.6485 PMC386528

[6] Levi-Schaffer F, Austen KF, Caulfield JP, Hein A, Gravallese PM, et al (1987) Co-culture of human lung-derived mast cells with mouse 3T3 fibroblasts: morphology and IgE-mediated release of histamine, prostaglandin D2, and leukotrienes. J Immunol 139: 494–500. 2439587

[7] Koma Y, Ito A, Watabe K, Hirata T, Mizuki M, et al (2005) Distinct role for c-kit receptor tyrosine kinase and SgIGSF adhesion molecule in attachment of mast cells to fibroblasts. Lab Invest 85: 426–435. 15654360 10.1038/labinvest.3700231

[8] Anderson DM, Lyman SD, Baird A, Wignall JM, Eisenman J, et al (1990) Molecular cloning of mast cell growth factor, a hematopoietin that is active in both membrane bound and soluble forms. Cell 63: 235–243. 1698558 10.1016/0092-8674(90)90304-w

[9] Flanagan JG, Chan DC, Leder P (1991) Transmembrane form of the kit ligand growth factor is determined by alternative splicing and is missing in the Sld mutant. Cell 64: 1025–1035. 1705866 10.1016/0092-8674(91)90326-t

[10] Dayton ET, Caulfield JP, Hein A, Austen KF, Stevens RL (1989) Regulation of the growth rate of mouse fibroblasts by IL-3-activated mouse bone marrow-derived mast cells. J Immunol 142: 4307–4313. 2786027

[11] Nigrovic PA, Lee DM (2007) Synovial mast cells: role in acute and chronic arthritis. Immunol Rev 217: 19–37. 17498049 10.1111/j.1600-065X.2007.00506.x

[12] Schmitz J, Owyang A, Oldham E, Song Y, Murphy E, et al (2005) IL-33, an interleukin-1-like cytokine that signals via the IL-1 receptor-related protein ST2 and induces T helper type 2-associated cytokines. Immunity 23: 479–490. 16286016 10.1016/j.immuni.2005.09.015

[13] Lunderius-Andersson C, Enoksson M, Nilsson G (2012) Mast Cells Respond to Cell Injury through the Recognition of IL-33. Front Immunol 3: 82. 22566963 10.3389/fimmu.2012.00082 PMC3342375

[14] Liew FY, Pitman NI, McInnes IB (2010) Disease-associated functions of IL-33: the new kid in the IL-1 family. Nat Rev Immunol 10: 103–110. 20081870 10.1038/nri2692

[15] Hsu CL, Neilsen CV, Bryce PJ (2010) IL-33 is produced by mast cells and regulates IgE-dependent inflammation. PLoS One 5: e11944. 20689814 10.1371/journal.pone.0011944 PMC2914748

[16] Tominaga S (1989) A putative protein of a growth specific cDNA from BALB/c-3T3 cells is highly similar to the extracellular portion of mouse interleukin 1 receptor. FEBS Lett 258: 301–304. 2532153 10.1016/0014-5793(89)81679-5

[17] Allakhverdi Z, Smith DE, Comeau MR, Delespesse G (2007) Cutting edge: The ST2 ligand IL-33 potently activates and drives maturation of human mast cells. J Immunol 179: 2051–2054. 17675461 10.4049/jimmunol.179.4.2051

[18] Ho LH, Ohno T, Oboki K, Kajiwara N, Suto H, et al (2007) IL-33 induces IL-13 production by mouse mast cells independently of IgE-FcepsilonRI signals. J Leukoc Biol 82: 1481–1490. 17881510 10.1189/jlb.0407200

[19] Iikura M, Suto H, Kajiwara N, Oboki K, Ohno T, et al (2007) IL-33 can promote survival, adhesion and cytokine production in human mast cells. Lab Invest 87: 971–978. 17700564 10.1038/labinvest.3700663

[20] Moulin D, Donze O, Talabot-Ayer D, Mezin F, Palmer G, et al (2007) Interleukin (IL)-33 induces the release of pro-inflammatory mediators by mast cells. Cytokine 40: 216–225. 18023358 10.1016/j.cyto.2007.09.013

[21] Xu D, Jiang HR, Kewin P, Li Y, Mu R, et al (2008) IL-33 exacerbates antigen-induced arthritis by activating mast cells. Proc Natl Acad Sci U S A 105: 10913–10918. 18667700 10.1073/pnas.0801898105 PMC2491487

[22] Enoksson M, Lyberg K, Moller-Westerberg C, Fallon PG, Nilsson G, et al (2011) Mast cells as sensors of cell injury through IL-33 recognition. J Immunol 186: 2523–2528. 21239713 10.4049/jimmunol.1003383

[23] Pushparaj PN, Tay HK, H’Ng SC, Pitman N, Xu D, et al (2009) The cytokine interleukin-33 mediates anaphylactic shock. Proc Natl Acad Sci U S A 106: 9773–9778. 19506243 10.1073/pnas.0901206106 PMC2700978

[24] Silver MR, Margulis A, Wood N, Goldman SJ, Kasaian M, et al (2009) IL-33 synergizes with IgE-dependent and IgE-independent agents to promote mast cell and basophil activation. Inflamm Res 59: 207–218. 19763788 10.1007/s00011-009-0088-5

[25] Andrade MV, Iwaki S, Ropert C, Gazzinelli RT, Cunha-Melo JR, et al (2010) Amplification of cytokine production through synergistic activation of NFAT and AP-1 following stimulation of mast cells with antigen and IL-33. Eur J Immunol 41: 760–772. 10.1002/eji.201040718 PMC3085255 21308681

[26] Kaieda S, Shin K, Nigrovic PA, Seki K, Lee RT, et al (2010) Synovial fibroblasts promote the expression and granule accumulation of tryptase via interleukin-33 and its receptor ST-2 (IL1RL1). J Biol Chem 285: 21478–21486. 20427273 10.1074/jbc.M110.114991 PMC2898446

[27] Thakurdas SM, Melicoff E, Sansores-Garcia L, Moreira DC, Petrova Y, et al (2007) The mast cell-restricted tryptase mMCP-6 has a critical immunoprotective role in bacterial infections. J Biol Chem 282: 20809–20815. 17456473 10.1074/jbc.M611842200

[28] McNeil HP, Shin K, Campbell IK, Wicks IP, Adachi R, et al (2008) The mouse mast cell-restricted tetramer-forming tryptases mouse mast cell protease 6 and mouse mast cell protease 7 are critical mediators in inflammatory arthritis. Arthritis Rheum 58: 2338–2346. 18668540 10.1002/art.23639

[29] Shin K, Nigrovic PA, Crish J, Boilard E, McNeil HP, et al (2009) Mast cells contribute to autoimmune inflammatory arthritis via their tryptase/heparin complexes. J Immunol 182: 647–656. 19109198 10.4049/jimmunol.182.1.647 PMC2610352

[30] Palmer G, Talabot-Ayer D, Lamacchia C, Toy D, Seemayer CA, et al (2009) Inhibition of interleukin-33 signaling attenuates the severity of experimental arthritis. Arthritis Rheum 60: 738–749. 19248109 10.1002/art.24305

[31] Xu D, Jiang HR, Li Y, Pushparaj PN, Kurowska-Stolarska M, et al (2010) IL-33 exacerbates autoantibody-induced arthritis. J Immunol 184: 2620–2626. 20139274 10.4049/jimmunol.0902685

[32] Binstadt BA, Patel PR, Alencar H, Nigrovic PA, Lee DM, et al (2006) Particularities of the vasculature can promote the organ specificity of autoimmune attack. Nat Immunol 7: 284–292. 16444258 10.1038/ni1306

[33] Nigrovic PA, Malbec O, Lu B, Markiewski MM, Kepley C, et al (2010) C5a receptor enables participation of mast cells in immune complex arthritis independently of Fcgamma receptor modulation. Arthritis Rheum 62: 3322–3333. 20662064 10.1002/art.27659 PMC2970731

[34] Smith DE (2011) The biological paths of IL-1 family members IL-18 and IL-33. J Leukoc Biol 89: 383–392. 20952658 10.1189/jlb.0810470

[35] Nigrovic PA, Binstadt BA, Monach PA, Johnsen A, Gurish M, et al (2007) Mast cells contribute to initiation of autoantibody-mediated arthritis via IL-1. Proc Natl Acad Sci U S A 104: 2325–2330. 17277081 10.1073/pnas.0610852103 PMC1892913

[36] Townsend MJ, Fallon PG, Matthews DJ, Jolin HE, McKenzie AN (2000) T1/ST2-deficient mice demonstrate the importance of T1/ST2 in developing primary T helper cell type 2 responses. J Exp Med 191: 1069–1076. 10727469 10.1084/jem.191.6.1069 PMC2193113

[37] Lee DM, Friend DS, Gurish MF, Benoist C, Mathis D, et al (2002) Mast cells: a cellular link between autoantibodies and inflammatory arthritis. Science 297: 1689–1692. 12215644 10.1126/science.1073176

[38] Lee DM, Kiener HP, Agarwal SK, Noss EH, Watts GF, et al (2007) Cadherin-11 in synovial lining formation and pathology in arthritis. Science 315: 1006–1010. 17255475 10.1126/science.1137306

[39] Razin E, Ihle JN, Seldin D, Mencia-Huerta JM, Katz HR, et al (1984) Interleukin 3: A differentiation and growth factor for the mouse mast cell that contains chondroitin sulfate E proteoglycan. J Immunol 132: 1479–1486. 6198393

[40] Kim ND, Chou RC, Seung E, Tager AM, Luster AD (2006) A unique requirement for the leukotriene B4 receptor BLT1 for neutrophil recruitment in inflammatory arthritis. J Exp Med 203: 829–835. 16567386 10.1084/jem.20052349 PMC2118298

[41] Alves-Filho JC, Sonego F, Souto FO, Freitas A, Verri WA Jr, et al (2010) Interleukin-33 attenuates sepsis by enhancing neutrophil influx to the site of infection. Nat Med 16: 708–712. 20473304 10.1038/nm.2156

[42] Wipke BT, Allen PM (2001) Essential role of neutrophils in the initiation and progression of a murine model of rheumatoid arthritis. J Immunol 167: 1601–1608. 11466382 10.4049/jimmunol.167.3.1601

[43] Jacobs JP, Ortiz-Lopez A, Campbell JJ, Gerard CJ, Mathis D, et al (2010) Deficiency of CXCR2, but not other chemokine receptors, attenuates autoantibody-mediated arthritis in a murine model. Arthritis Rheum 62: 1921–1932. 20506316 10.1002/art.27470 PMC2994550

[44] Shushakova N, Skokowa J, Schulman J, Baumann U, Zwirner J, et al (2002) C5a anaphylatoxin is a major regulator of activating versus inhibitory FcgammaRs in immune complex-induced lung disease. J Clin Invest 110: 1823–1830. 12488432 10.1172/JCI200216577 PMC151656

[45] Ohno T, Oboki K, Morita H, Kajiwara N, Arae K, et al (2011) Paracrine IL-33 stimulation enhances lipopolysaccharide-mediated macrophage activation. PLoS One 6: e18404. 21494550 10.1371/journal.pone.0018404 PMC3073971

[46] Galli SJ, Maurer M, Lantz CS (1999) Mast cells as sentinels of innate immunity. Curr Opin Immunol 11: 53–59. 10047539 10.1016/s0952-7915(99)80010-7

[47] Chou RC, Kim ND, Sadik CD, Seung E, Lan Y, et al (2010) Lipid-cytokine-chemokine cascade drives neutrophil recruitment in a murine model of inflammatory arthritis. Immunity 33: 266–278. 20727790 10.1016/j.immuni.2010.07.018 PMC3155777

[48] Blatner NR, Bonertz A, Beckhove P, Cheon EC, Krantz SB, et al (2010) In colorectal cancer mast cells contribute to systemic regulatory T-cell dysfunction. Proc Natl Acad Sci U S A 107: 6430–6435. 20308560 10.1073/pnas.0913683107 PMC2851977

[49] Komai-Koma M, Brombacher F, Pushparaj PN, Arendse B, McSharry C, et al.. (2012) Interleukin-33 amplifies IgE synthesis and triggers mast cell degranulation via interleukin-4 in naive mice. Allergy. 10.1111/j.1398-9995.2012.02859.x PMC3660789 22702477

[50] Wang JX, Bair AM, King SL, Shnayder R, Huang YF, et al (2012) Ly6G ligation blocks recruitment of neutrophils via a beta 2 integrin-dependent mechanism. Blood 120: 1489–98. 22661700 10.1182/blood-2012-01-404046 PMC3423786

[51] Luthi AU, Cullen SP, McNeela EA, Duriez PJ, Afonina IS, et al (2009) Suppression of interleukin-33 bioactivity through proteolysis by apoptotic caspases. Immunity 31: 84–98. 19559631 10.1016/j.immuni.2009.05.007

[52] Kakkar R, Hei H, Dobner S, Lee RT (2012) Interleukin 33 as a mechanically responsive cytokine secreted by living cells. J Biol Chem 287: 6941–8. 22215666 10.1074/jbc.M111.298703 PMC3307313

[53] Nigrovic PA, Lee DM (2006) Immune complexes and innate immunity in rheumatoid arthritis. In: Firestein GS, Panayi GS, Wollheim FA, editors. Rheumatoid Arthritis: new frontiers in pathogenesis and treatment. 2nd ed. Oxford: Oxford University Press. 135–156.

